# Hesitancy towards Childhood Vaccinations among Parents of Children with Underlying Chronic Medical Conditions in Italy

**DOI:** 10.3390/vaccines10081254

**Published:** 2022-08-04

**Authors:** Francesco Napolitano, Grazia Miraglia del Giudice, Silvia Angelillo, Italo Fattore, Francesca Licata, Concetta Paola Pelullo, Gabriella Di Giuseppe

**Affiliations:** 1 Department of Experimental Medicine, University of Campania “Luigi Vanvitelli”, 80138 Naples, Italy; 2 Department of Health Sciences, University of Catanzaro ‘‘Magna Græcia”, 88100 Catanzaro, Italy; 3 Department of Movement Sciences and Wellbeing, University of Naples “Parthenope”, 80133 Naples, Italy

**Keywords:** children, chronic medical conditions, cross-sectional study, Italy, PACV, vaccine hesitancy

## Abstract

Background: This study was designed to evaluate vaccination hesitancy and behaviors among parents of children with chronic conditions. Methods: This cross-sectional study was conducted from June to December 2021 in three public hospitals in southern Italy. Data were collected using a face-to-face interview of parents of children up to 17 years of age with at least one chronic condition. Results: Of the 532 parents approached, 444 agreed to participate, with a response rate of 83.4%. Almost half of parents (43%) knew that children with chronic diseases are at greater risk of complications from VPDs, and 21.6% knew all the vaccinations available in Italy. Additionally, 55.9% felt that vaccine-preventable diseases (VPDs) are very dangerous for their children, and 28.7% were very worried about the side effects of vaccines. The result of the Parent Attitudes about Childhood Vaccine (PACV) score indicated that 23.2% of parents were hesitant about vaccinations. Parental vaccine hesitancy was significantly more common among parents who had female children, among those who did not know the recommended vaccinations, among those who had a higher concern of potential side effects of the vaccines, among those who believed that the administration of the vaccinations was not useful, and among who received information on recommended vaccination from the internet, social and mass media. Conclusions: Important efforts by policy makers and healthcare providers must be implemented to counter vaccine hesitancy among parents.

## 1. Introduction

It is well known that the implementation of immunization programs worldwide has led to a reduction in the incidence of vaccine-preventable diseases (VPDs), with a consequent decrease in severe complications, disability, and mortality associated with VPDs, as well as saving health resources, in particular for the groups of patients at high risk, such as children and adults with chronic conditions [1].

In Italy, ten vaccinations (against tetanus, diphtheria, pertussis, poliomyelitis, *Haemophilus influenzae* type b, hepatitis B, measles, mumps, rubella, and varicella) have been mandatory during childhood since 2017, whereas vaccinations against meningococcal disease, pneumococcal disease, rotavirus, and seasonal influenza, are recommended during childhood or to patients with chronic conditions, and are provided free of charge by primary care pediatricians or vaccination centers in Italian Local Health Units [2]. Although the mandatory vaccination has led to an increase in coverage against several infectious diseases during childhood, the recommended vaccinations are still far from reaching the adequate coverage, and this is alarming, especially for children with chronic conditions [3,4,5]. Therefore, planning educational interventions among parents and promoting vaccinations of children with chronic conditions such as respiratory, cardiovascular, renal, and neoplastic diseases are fundamental strategies, and represent a priority objective of public health to improve the coverage and to prevent and limit the spread of the infectious diseases in this population group.

Among barriers to vaccine administration, parents’ hesitancy about childhood vaccinations has been well documented in several investigations worldwide, with the rate of hesitant parents measured with different methodologies ranging from 8.2% to 45.2% [6,7,8,9,10,11], whereas previous studies conducted in Italy showed that the proportion of hesitant parents ranged from 7.7% to 34.7% [12,13,14,15,16,17]. Moreover, it was also confirmed that vaccine hesitancy may negatively affect the immunization rate in several populations [18,19,20,21,22], with an increased risk of spread of VPDs among children [23,24,25].

Several studies have investigated the determinants that may influence parents’ vaccination behaviors, such as concerns about vaccine side effects, hesitancy and lack of trust in vaccination, and difficulty accessing healthcare facilities, whereas a gap in knowledge in the literature was found in Italy regarding parents’ knowledge, behaviors, and hesitancy about vaccinations for children with underlying chronic medical conditions [14,15,26,27]. The results of studies carried out on parents of children with chronic diseases could be used to implement vaccination strategies to effectively increase coverage in this at-risk group and avoid the health complications of VPDs.

Therefore, this study was designed to evaluate vaccination hesitancy and behaviors and to investigate the relative determinants among parents of children with underlying chronic medical conditions in Italy.

## 2. Materials and Methods

### 2.1. Study Population and Sampling

This cross-sectional study was conducted from June to December 2021 in two randomly selected public hospitals of the geographic area of two provinces in Campania (Naples and Caserta) and one randomly selected public hospital in Calabria (Catanzaro), Italy. The target population was composed of parents of children up to 17 years of age with at least one chronic condition. Study participants were randomly approached in the waiting room before their child’s scheduled visit in the following hospital ambulatory centers: diabetic, hemato-oncology, endocrinology, nephrology, rheumatology, allergy, and clinical immunology. Only one parent per child was interviewed. A minimum target sample size of 403 was estimated, assuming that 30% of parents were hesitant according to previously published literature [15], with a margin error of 5%, a confidence interval of 95%, and considering a response rate of 80%.

After obtaining approval from the Ethics Committee of the Teaching Hospital of the University of Campania “Luigi Vanvitelli” (protocol number: 8666/2021), the directors of the selected hospitals received a letter explaining the purpose of the investigation and the procedure of the survey and requesting their collaboration.

After the approval, experienced trained physicians not involved in the clinical care approached the participants, informed the selected parents about the study objectives and methodology, and assured them that the study participation was voluntary, that all information would remain confidential and would be analyzed anonymously, and that they had the right to refuse or to withdraw at any moment from the interview without disclosing any reason. No incentives and gift were offered to parents.

A pilot study was performed among 25 parents (included in the final sample) to evaluate the comprehensibility and the validity of the questions, and no changes were made to the survey instrument.

### 2.2. Survey Instrument

The research team designed the questionnaire used to collect data based on previous studies conducted in several groups of population about the hesitancy and practices regarding vaccinations [15,17,28]. A copy of the questionnaire is provided as an additional file (Supplementary File S1).

The questionnaire consisted of 40 questions divided into four major sections. The first section investigated the socio-demographic characteristics of the parents and children (gender, age, marital status, number of children, level of education and parents’ working activity, chronic medical condition of children, pharmacological therapy, and parents’ perception of their health status). The second section was designed to assess the parents’ vaccination knowledge about the immunization program for their children in Italy, regarding the mandatory (against tetanus, diphtheria, pertussis, poliomyelitis, *Haemophilus influenzae* type b, hepatitis B, measles, mumps, rubella, and varicella) and recommended vaccinations (against meningococcal disease, pneumococcal disease, rotavirus, Human Papillomavirus (HPV), and seasonal influenza) that were provided free of charge by primary care pediatricians or vaccination centers in Italian Local Health Units. The possible answers were “no”, “do not know”, and “yes”. In the third section, parents were asked about their attitude regarding childhood vaccinations (perceived severity of VPDs; vaccines’ effectiveness and safety). The attitudes were measured on a ten-point Likert Scale ranging from 1 representing “not at all” to 10 representing “at all”. Parents were also asked if they delayed or refused at least one shot of vaccinations for their children, and the reasons for having delayed or refused the vaccines. Parental vaccine hesitancy was assessed using the 15-item Parent Attitudes about Childhood Vaccine (PACV) survey translated into the Italian language, which had already been used in previous published studies conducted by the research team [15,17,29]. The PACV consists of three domains: vaccine behavior, beliefs about vaccine safety and efficacy, and general attitude and trust. The score ranged from 0 to 100 and a parent was defined as hesitant if the score was ≥50.

The last section was aimed to collect data on information sources about vaccinations and if the parents perceived a need for additional information.

### 2.3. Statistical Analysis

First, descriptive analysis, including means and standard deviations for continuous variables and proportions for categorical variables, was used to describe the main parents’ characteristics. Second, bivariate analysis was performed to assess the association between each of the independent characteristics and the different outcomes of interest, using the chi-square test for the categorical variables and Student’s *t*-test for the continuous variables. The variables with a *p*-value < 0.25 in bivariate analysis were included in multivariate linear and logistic regression models. Three multivariate models were designed to address the possible association between the different variables and the following dependent variables: considering the recommended vaccinations very dangerous for their children (continuous) (Model 1); having refused or delayed at least one shot of the recommended vaccinations for their children with chronic conditions (no = 0; yes = 1) (Model 2); and parents’ vaccine hesitancy (PACV score <50 = 0; PACV score ≥50 = 1) (Model 3). The following independent variables were included in all Models: age in years (continuous), gender (male = 0; female = 1), marital status (unmarried/separated/divorced/widowed = 0; married/cohabiting with a partner = 1), baccalaureate/graduate degree (no = 0; yes = 1), at least one parent being a healthcare professional (no = 0; yes = 1), having more than one child (no = 0; yes = 1), age of the children with chronic conditions (continuous, in years), gender of the children with chronic conditions (male = 0; female = 1), children taking medications (no = 0; yes = 1), parents’ perceived health status of the children with chronic conditions (continuous), knowledge about the vaccinations available in Italy (no = 0; yes = 1), knowledge about the recommended vaccinations for their children with chronic conditions (no = 0; yes = 1), perceived the VPDs as very dangerous for their children with chronic conditions (continuous), believing that the administration of the vaccinations is useful for their children with chronic conditions (no = 0; yes = 1), and need for additional information about vaccinations (no = 0; yes = 1). The following variables were also included in the different models: knowledge that children with chronic diseases are at greater risk of complications from VPDs (no = 0; yes = 1) in Model 1; considering the recommended vaccinations very dangerous for their children (continuous) in Model 2 and 3; having received information about the recommended vaccinations for children with chronic conditions from physicians (no = 0; yes = 1) in Model 1; having received information about the recommended vaccinations for children with chronic conditions from internet/mass media/social media (n = 0; yes = 1) in Model 2 and 3; having had discussions with pediatricians/medical specialists about the recommended vaccinations for their children with chronic conditions (no = 0; yes = 1) in Model 2 and 3. Values of *p* = 0.2 and *p* = 0.4 were used to select candidate variables for retention and exclusion in the final multivariate models. Results of the logistic regression models were measured using Odds Ratios (ORs) with 95% confidence intervals (CIs), whereas results of the linear regression models used standardized regression coefficients (ß). For all analyses, two-tailed tests were used and a *p*-value equal to or less than 0.05 was considered statistically significant. Stata statistical software version 15.1 was used to analyze the data [30].

## 3. Results

### 3.1. Characteristics of Study Population

Of the 532 parents approached, 444 agreed to participate, with a response rate of 83.4%. The main characteristics of the study population were described in Table 1. More than two-thirds of the parents were female (87.8%) and married/cohabited with a partner (91.2%), the average age was 40.1 years (range 20–63 years), a quarter had a laureate/baccalaureate degree (26.8%), nearly half were employed (48.8%), and more than two-thirds had more than one child (77.7%). Regarding the sample of children with chronic conditions, the mean age was 9.6 years, more than half (55.1%) were male, the most prevalent chronic medical conditions encountered were kidney diseases (21.6%), diabetes (21.2%), endocrinologic diseases (18.2%), and autoimmune diseases (9%), and the mean value of the perceived health status of children reported by the parents was 8.8, on a scale ranging from 1 to 10.

### 3.2. Parents’ Knowledge about Vaccinations

Almost half of parents (43%) knew that children with chronic diseases are at greater risk of complications from VPDs, and approximately one in five parents (21.6%) knew all the vaccinations available in Italy, with a better knowledge found for measles, mumps, and rubella vaccines (94.4%) and the influenza vaccine (93%), whereas 82% of parents knew of the HPV vaccination and only 55.6% were aware of the availability of the vaccine against rotavirus.

When asked about the recommended vaccinations for children, two-thirds of parents knew that pneumococcal (69.7%) and influenza (69.1%) vaccines are recommended for children with chronic conditions, 58.3% of participants indicated the rotavirus vaccination, and more than half (56.3%) correctly knew the vaccination recommendation against meningococcal disease. Moreover, 63.5% of parents knew all the mandatory vaccinations during childhood and adolescence in Italy.

### 3.3. Parents’ Hesitancy and Behaviors about Vaccinations

The majority of parents (55.9%) felt that VPDs are very dangerous for their children with chronic conditions, with a mean value of 8.6, on a scale ranging from 1 to 10, and 28.7% were very worried about the side effects of the vaccines. More than half of the participants (53.2%) believed that the administration of the vaccinations is useful for their children, with a mean value of 8.6 on a scale ranging from 1 to 10, whereas one in four (26.7%) considered the recommended vaccinations very dangerous for their children, with an average value of 6.3. The results of multivariate linear regression analysis showed that this negative attitude was more likely among parents without a baccalaureate/graduate degree, among those who do not work as a healthcare professional, among those who knew that children with chronic diseases are at greater risk of complications from VPDs, and among those who do not believe that the administration of the vaccinations is useful for their children (Model 1 in Table 2).

Only 11.9% and 11% of parents had declared that they delayed and refused at least one shot of a vaccine for their children with chronic conditions, respectively. The most frequent reasons for delay were difficulties due to the COVID-19 pandemic (40%), forgetfulness (22%), fear of vaccines’ side effects (16%), and the lack of recommendation by pediatricians (6%). Moreover, the most frequent reasons of those refusing were fear of vaccines’ side effects (58.7%), not considering the recommended vaccinations useful (24%), and the lack of recommendation by pediatricians (6.5%). The results of the multivariate logistic regression analysis showed that parents who had not discussed with pediatricians/medical specialists on the recommended vaccinations for their children with chronic conditions (OR = 0.47; 95% CI = 0.22–0.99), and those who had children who took medications for their chronic conditions (OR = 2.06; 95% CI = 1.03–4.13) were more likely to have refused at least one shot of a vaccine for their children (Model 2 in Table 2).

The result of the PACV score indicated that 23.2% of parents were hesitant about vaccinations. The parents’ responses for each item on the PACV are shown in Table 3. In particular, one in four (23.9%) agree strongly/agree that children get more shots than are good for them, and more than two-thirds (70.5%) agreed/strongly agreed that many of the infectious diseases shots prevent are severe. Almost half of parents (48.6%) were very worried that their children would have a serious vaccine side effect and 36.8% that vaccines could be unsafe. More than two-thirds (70.3%) agreed/strongly agreed that they trust the information they received on vaccination, and a large majority (87.8%) agreed/strongly agreed that they could openly discuss their concerns about vaccinations with their pediatricians. Moreover, the parents’ self-reported trust in their pediatricians had a mean value of 8.6, on a scale ranging from 1 to 10.

A multivariate logistic regression model built to identify the factors associated with parental vaccine hesitancy showed that the hesitancy was significantly more common among parents who had female children (OR = 2.1; 95% CI = 1.18–3.73), among those who did not know the recommended vaccinations (OR = 0.27; 95% CI = 0.15–0.48), among those who had a higher concern for potential side effects of the vaccines on their children with chronic conditions (OR = 1.33; 95% CI = 1.19–1.49), among those who believed that the administration of the vaccinations was not useful (OR = 0.61; 95% CI = 0.52–0.71), and among those who received information on recommended vaccinations via the internet, social and mass media (OR = 1.87; 95% CI = 1.05–3.33) (Model 3 in Table 2).

A large majority of parents declared that they had discussions with pediatricians/medical specialists regarding the recommended vaccinations for their children with chronic conditions (87.4%) and, when parents were asked about the vaccination coverages of children, 94.6% self-reported that their children had received the current mandatory vaccinations in Italy (tetanus, diphtheria, pertussis, *Haemophilus influenzae* type b, hepatitis B, poliomyelitis, varicella, measles, mumps, and rubella vaccines); only 8.3% and 4.5% declared that their children had received vaccination against pneumococcal disease and rotavirus, respectively, and no parent reported that their children had received vaccination against meningococcal disease. Moreover, only 10.6% of parents indicated that their child had been vaccinated against seasonal influenza in the last year, and only 28.5% of parents of boys/girls aged ≥12 years had immunized their children against HPV.

### 3.4. Sources of Information

Only 1.3% of parents reported no source of information on vaccinations; a large majority of parents (82.6%) reported pediatricians/medical specialists as their main source of information on recommended vaccinations for their children with chronic conditions, and other sources of knowledge were the internet (33.8%), mass media (29%), and social media (20%). One-third (34.7%) of participants expressed the need for more information about the vaccinations.

## 4. Discussion

This study provides relevant information about the hesitancy and behaviors towards vaccinations among parents of children with chronic conditions in Italy and the relative determining factors.

Regarding the parents’ knowledge of vaccinations, it is important to underline that not even half of the parents who participated in the study knew that children with chronic diseases are at greater risk of complications from VPDs, and only one in five knew all the vaccinations available in Italy for their children. These results are very alarming, as correct awareness of the risk of contracting infectious diseases and correct knowledge of parents about the available vaccinations are a fundamental prerequisite for being able to effectively implement all vaccination coverage strategies during childhood and adolescence. In particular, healthcare professionals should spend more time correctly informing the parents of the benefit of vaccinations, as in our study, knowledge about vaccinations is quite low, despite self-reported parents’ trust in the pediatricians reaching a high value and pediatricians/medical specialists being the main sources of knowledge on vaccinations reported by parents.

Regarding the parents’ attitudes and behaviors about vaccinations, a positive attitude was found regarding the dangerousness of VPDs and the usefulness of vaccinations, even if one-third of parents were very concerned about the vaccines’ side effects. The fear of side effects in this study is also the most frequent reason of those refusing at least one shot of a vaccine for their children with chronic conditions, and it has been reported in previous studies in the literature as an important barrier for vaccine uptake [14,15,17,21,28,29,31]. Several interventions, such as individually tailored education, vaccine information pamphlets, and specialist immunization clinics, focused on vaccine safety have been shown to be effective in increasing confidence in vaccination, the intention to vaccinate, and vaccination uptake [32], and these should be implemented in different care settings by health policy makers, healthcare workers, and public health experts to counteract the vaccine hesitancy. Instead, difficulties due to the COVID-19 pandemic are the most frequent reason of those delaying at least one shot of vaccine for their children with chronic conditions. This reason for delaying vaccinations that put many children at risk of infectious diseases can be explained by considering both the disruptive impact that the COVID-19 pandemic has had on the operations of public vaccination centers in many countries, especially at the beginning (March–April 2020) of the spread of the SARS-CoV-2 infection in Italy; the restrictive measures put in place by the Italian government; and the fear of acquiring the SARS-CoV-2 infection in healthcare facilities [33,34]. Moreover, among the main reasons for refusing and delaying vaccinations, parents also indicated the lack of recommendation by the pediatricians, and the results of multivariate logistic regression analysis showed that parents who had not received information from pediatricians on the recommended vaccinations for their children with chronic conditions were more likely to have refused at least one shot of vaccine for them. Therefore, these results recall the urgent need for more effective educational intervention on vaccination and a dissemination program regarding the value and safety of vaccines towards the parents, which must see primary care pediatricians and medical specialists involved as protagonists. All occasions and setting in which it is possible to meet parents and at-risk children, such as parents’ access to primary care pediatrician’s clinics, to ambulatory centers in Hospitals and Local Health Units, and to vaccinations centers should be leveraged to implement healthcare recommendations about vaccination and to improve the coverage. Indeed, vaccine recommendation by healthcare workers (HCWs) was found in previous investigations to be a strong predictor of vaccinations uptake during childhood and in other at-risk groups [35,36,37,38,39].

The results of the PACV score indicated that the 23.2% of parents were hesitant about childhood vaccinations. It is important to take into account that this result may be influenced by the fact that this study has been performed during the COVID-19 pandemic. Indeed, a recent review showed that the COVID-19 vaccine hesitancy rate in Italy is 59.9% [40], and this may have contributed to increasing negative attitudes towards other vaccinations. The comparison of the results of this study with previous investigations conducted using PACV score showed that lower values of parental vaccine hesitancy have been observed in Greece during the COVID-19 era (8.9%) [41], as well as in Ireland (6.7% and 14.4%) [42,43], Italy (7.7%) [14], and Canada (15%) [44] prior to the COVID-19 pandemic, whereas parental vaccine hesitancy in this study was lower compared to the value reported in a previous investigation conducted by some of us in the same geographical area (34.7%) [15] and in the US (26%) [45] before the spread of SARS-CoV-2 infection. Moreover, a recent study conducted in Italy showed that 26.3% of parents were highly hesitant about the COVID-19 vaccine for their children with chronic conditions [46]. The difference in methodology and findings of the studies on parental vaccine hesitancy conducted pre- and post-COVID-19 pandemic and the complexity of the reasons why people choose to refuse or delay the vaccinations suggest that further investigations are needed to establish whether vaccine hesitancy have been increased by the pandemic. However, it should be emphasized that in this study, hesitancy is even more worrying because it affects parents of children with chronic diseases who are more susceptible to the complications of VPDs. Healthcare professionals should have more focus on this at-risk group by providing the correct information to raise parents’ awareness of vaccine efficacy and the risk of VPDs. Moreover, they should be more careful in vaccine recommendations and in verifying that children have been immunized.

The results of the multivariate regression analysis showed that parental vaccine hesitancy was more likely among those who considered the recommended vaccinations dangerous for their children and among those who received information on recommended vaccination via the internet, social and mass media. This result is alarming, as it is known that internet use and social networks can spread false and misleading information on vaccinations and can increase parents’ concerns about vaccine safety and negatively affect the coverage rates [47]. Thus, it is important that healthcare providers understand the population’s knowledge needs regarding vaccinations and health policy makers monitor and counter the spread of fake news about vaccinations on the internet and social media, promoting trust in the evidence-based information available on health authority websites.

In our sample, despite a large majority that reported having discussions with pediatricians/medical specialists about the recommended vaccinations for their children with chronic conditions, inadequate coverage rates were observed for the recommended vaccines, with only a very small proportion of the parents reported having vaccinated their children against pneumococcal disease, rotavirus infection, and seasonal influenza. These coverages are concerning, and have also been found in Italy at a national level [3], and these findings can be explained by the fact that the knowledge of the public on non-mandatory vaccinations is inadequate, and that public vaccination interventions and physicians’ recommendations to improve the coverage in at-risk groups of population are insufficient.

This investigation has several potential limitations due to study design and methodology that must be taken into consideration when analyzing the findings. The first is that it is not possible to establish the temporal direction of the association between the outcomes of interest and the influencing factors in the cross-sectional study. The second is that the data were collected through interviews, and it is possible that respondents may be influenced in their responses by the defined socially appropriate attitudes and behaviors, and this could overestimate the positive attitudes and behaviors towards vaccinations. To address this limitation, the interviews were carried out without reporting the information on the participant’s identification and the confidentiality of the answers was ensured. Third, there may be a recall bias that could lead to underestimating or overestimating vaccination coverage because parents were asked to self-report the children’s vaccination status. Despite these limitations, the sample size was appropriate, with a high response rate, and the results of this survey provided important information on parental hesitancy towards vaccinations of children with underlying chronic medical conditions in Italy.

## 5. Conclusions

In conclusion, important efforts by policy makers and healthcare providers must be implemented to counter vaccine hesitancy among parents of children with chronic conditions. Therefore, educational interventions aimed at addressing the concerns about vaccinations should be more widely carried out, as the fear of the adverse effects of vaccines is the primary concern of parents in this study. The role of pediatricians and specialists as a source of information and in administering vaccines must be strengthened within immunization programs, given the trust they have among parents and given that the study findings had showed that many parents are exposed to vaccine information on the internet and social media, and this can lead to misinformation and mistrust. Therefore, the vaccinations recommendation of pediatricians and medical specialists is a key factor for decision making of parents regarding vaccination in order to improve their knowledge of vaccines, and it is pivotal to increase the coverage rates, as the results of our survey showed that the coverage for recommended vaccinations in Italy is worryingly insufficient. Further investigations are needed to explore the attitudes and behaviors of HCWs regarding recommended vaccinations for children with chronic conditions.

## Figures and Tables

**Table 1:** Socio-demographic and key characteristics of the study population.

Parents’ Characteristics	Total		Vaccine Hesitant		Non Vaccine Hesitant	
	N	%	N	%	N	%
Age, years	40.1 ± 7.4 (20–63) *		40.3 ± 7 (23–58) *		40.1 ± 7.52 (20–63) *	
			*t* = −0.28; *p* = 0.776			
Gender						
Male	54	12.2	12	22.2	42	77.8
Female	390	87.8	91	23.3	299	76.7
			χ^2^ = 0.033; *p* = 0.856			
Marital status						
Married/cohabited with a partner	404	91.2	93	23	311	77
Unmarried/separated/divorced/widowed	39	8.8	10	25%	30	75
			χ^2^ = 0.08; *p* = 0.777			
Educational level						
High school degree or less	325	73.2	88	27.1	237	72.9
Baccalaureate/graduate degree	119	26.8	15	12.6	104	87.4
			χ^2^ = 10.24; *p* = 0.001			
Employment status						
Employed	217	48.8	37	17	180	83
Unemployed	227	51.1	66	29.1	161	70.9
			χ^2^ = 9; *p* = 0.003			
Number of children						
1	99	22.3	22	22.2	77	77.8
>1	345	77.7	81	23.5	264	76.5
			χ^2^ = 0.07; *p* = 0.794			
**Children’s characteristics**						
Age, years	9.6 ± 4.7 (1–17) *		10.6 ± 4.48 (1–17) *		9.3 ± 4.73 (1–17) *	
			*t* = −2.5; *p* = 0.0128			
Gender						
Male	244	55.1	48	19.7	196	80.3
Female	199	44.9	55	27.6	144	72.4
			χ^2^ = 3.89; *p* = 0.048			
Underlying chronic medical condition						
Kidney diseases	96	21.6	22	22.9	74	77.1
			χ^2^ = 0.005; *p* = 0.941			
Diabetes	94	21.2	26	27.7	68	72.3
			χ^2^ = 1.33; *p* = 0.248			
Endocrinologic diseases	81	18.2	18	22.2	63	77.8
			χ^2^ = 0.05; *p* = 0.818			
Autoimmune diseases	40	9	4	10	36	90
			χ^2^ = 4.29; *p* = 0.038			
Gastroenterological diseases	33	7.4	7	21.2	26	78.9
			χ^2^ = 0.08; *p* = 0.779			
Congenital diseases	32	7.2	4	12.5	28	87.5
			χ^2^ = 2.21; *p* = 0.137			
Rheumatologic diseases	31	7	7	22.6	24	77.4
			χ^2^ = 0.007; *p* = 0.933			
Onco-hematologic diseases	24	5.4	12	50	12	50
			χ^2^ = 10.23; *p* = 0.001			
Other	27	6.2				
Pharmacological therapy						
Yes	266	60.3	65	24.2	204	75.8
No	175	39.7	38	21.7	137	78.3
			χ^2^ = 0.36; *p* = 0.55			
Perceived health status of children reported by the parents	8.8 ± 1.6 (1–10) *		8.6 ± 1.48 (2–10) *		8.8 ± 1.64 (1–10) *	
			*t* = 0.9; *p* = 0.368			

Number for each item may not add up to total number of study population due to missing value. * Mean ± Standard deviation (range).

**Table 2:** Multivariate linear and logistic regression analyses indicating associations between several variables and the outcomes of interest.

Variable	Coeff.	SE	*t*	*p*
**Model 1.** Considering the recommended vaccinations very dangerous for their children F(12, 425) = 13.85, *p* < 0.0001, R^2^ = 28.1%, adjusted R^2^ = 26.1%				
Those who knew that children with chronic diseases are at greater risk of complications from VPDs	1.60	0.28	5.60	<0.001
Those who do not believe that the administration of vaccinations is useful for their children	−0.47	0.07	−6.36	<0.001
Not having a baccalaureate/graduate degree	−1.08	0.33	−3.24	0.001
Those who do not work as a healthcare professional	−1.38	0.59	−2.32	0.021
Unmarried/separated/divorced/widowed	−0.92	0.48	−1.91	0.057
Parents’ perceived health status of the children with chronic conditions	0.17	0.09	1.89	0.059
Female children	0.64	0.42	1.51	0.131
Not having received information about the recommended vaccinations for children with chronic conditions from physicians	−0.51	0.37	−1.39	0.164
No need for additional information about vaccinations	−0.41	0.30	−1.36	0.176
Those who do not know of all vaccinations available in Italy	−0.46	0.35	−1.33	0.183
Having children who take medications	0.37	0.29	1.26	0.207
Older children	0.03	0.03	1.19	0.236
**Variable**	**OR**	**SE**	**95% CI**	***p***
**Model 2.** Having refused or delayed at least one shot of a recommended vaccination for their children with chronic conditions Log likelihood =−149.64, χ^2^ = 24.41 (7 df), *p* = 0.0010				
Those who had children who took medications for their chronic conditions	2.06	0.73	1.03–4.13	0.041
Those who had not had discussions with pediatricians/medical specialists regarding the recommended vaccinations for their children with chronic conditions	0.47	0.18	0.22–0.99	0.049
Having more than one child	2.29	0.98	0.99–5.29	0.052
Younger children	0.93	0.03	0.87–1.00	0.063
Parents’ perceived health status of the children with chronic conditions	0.86	0.07	0.73–1.02	0.088
Having received information about the recommended vaccinations for children with chronic conditions from the internet/mass media/social media	1.69	0.53	0.92–3.12	0.092
Those who believed that the administration of the vaccinations is not useful for their children with chronic conditions	0.89	0.06	0.78–1.03	0.125
**Model 3.** Parental vaccine hesitancy Log likelihood =−157.67, χ^2^ = 161.15 (8 df), *p* < 0.0001				
Those who believed that the administration of the vaccinations is not useful for their children with chronic conditions	0.61	0.47	0.52–0.71	<0.001
Those who considered the recommended vaccinations very dangerous for their children	1.33	0.07	1.19–1.49	<0.001
Those who did not know the recommended vaccinations for their children with chronic conditions	0.27	0.08	0.15–0.48	<0.001
Female children	2.1	0.61	1.18–3.73	0.011
Having received information about the recommended vaccinations for children with chronic conditions from internet/mass media/social media	1.87	0.55	1.05–3.33	0.032
Not having a baccalaureate/graduate degree	0.65	0.24	0.31–1.35	0.247
Those who had not discussed with pediatricians/medical specialists regarding the recommended vaccinations for their children with chronic conditions	0.61	0.26	0.26–1.43	0.253
Older children	1.03	0.32	0.97–1.09	0.309

**Table 3:** Descriptive characteristics of PACV.

Item	Parent Response	N(%)
Have you ever delayed having your child get a shot for reasons other than illness or allergy?	Yes	53 (11.9)
	No	391 (88.1)
Have you ever decided not to have your child get a shot for reasons other than illness or allergy?	Yes	49 (11)
	No	395 (89)
How sure are you that following the recommended shot schedule is a good idea for your child?	0–10	8.4 ± 2 *
Children get more shots than are good for them.	Strongly agree/Agree	106(23.9)
	Strongly disagree/Disagree	277(62.4)
	Not sure	61(13.7)
I believe that many of the illnesses shots prevent are severe.	Strongly agree/Agree	313(70.5)
	Strongly disagree/Disagree	109(24.5)
	Not sure	22 (5)
It is better for my child to develop immunity by getting sick than to get a shot.	Strongly agree/Agree	107 (24.1)
	Strongly disagree/Disagree	285 (64.2)
	Not sure	52 (11.7)
It is better for children to get fewer vaccines at the same time.	Strongly agree/Agree	169 (38.1)
	Strongly disagree/Disagree	191 (43)
	Not sure	84 (18.9)
How concerned are you that your child might have a serious side effect from a shot?	Extremely concerned/Moderately concerned	324 (73)
	Slightly concerned/Not at all concerned	85 (19.1)
	Not sure	35 (7.9)
How concerned are you that any one of the childhood shots might not be safe?	Extremely concerned/Moderately concerned	275 (62.1)
	Slightly concerned/Not at all concerned	128 (28.9)
	Not sure	40 (9)
How concerned are you that a shot might not prevent the disease?	Extremely concerned/Moderately concerned	217 (48.8)
	Slightly concerned/Not at all concerned	182 (41)
	Not sure	45 (10.2)
If you had another infant today, would you want him/her to get all the recommended shots?	Yes	31 (7)
	No	366 (82.4)
	Do not know	47 (10.6)
Overall, how hesitant about childhood shots would you consider yourself to be?	Extremely hesitant/Moderately hesitant	122 (27.5)
	Slightly hesitant/Not at all hesitant	303 (68.2)
	Not sure	19 (4.3)
I trust the information I receive about shots.	Strongly agree/Agree	312 (70.3)
	Strongly disagree/Disagree	96 (21.6)
	Not sure	36 (8.1)
I am able to openly discuss my concerns about shots with my child’s doctor.	Strongly agree/Agree	387 (87.8)
	Strongly disagree/Disagree	39 (8.8)
	Not sure	15 (3.4)
All things considered, how much do you trust your child’s doctor?	0–10	8.6 ± 2.1*

Number for each item may not add up to total number of study population due to missing value. * Mean ± Standard deviation.

## Data Availability

The data presented in this study are available on request from the corresponding author.

## Supplementary Materials

The following supporting information can be downloaded at: https://www.mdpi.com/article/10.3390/vaccines10081254/s1. File S1. Questionnaire.
