# Supplementary material for: Hesitancy towards Childhood Vaccinations among Parents of Children with Underlying Chronic Medical Conditions in Italy
Source: Vaccines (Basel). 2022 Aug 4;10(8):1254. doi: 10.3390/vaccines10081254 (PMC9416633; doi:10.3390/vaccines10081254)
Supplement: Supplementary file 1 [file vaccines-10-01254-s001.zip › vaccines-1851904-supplementary.pdf]

QUESTIONNAIRE

A. SOCIO-DEMOGRAPHIC AND ANAMNESTIC CHARACTERISTICS

The questions in this section are aimed to acquire information on your socio-demographic and anamnestic characteristics

A1. How old are you? ________________

A2. What is your gender? □ Male □ Female

A3. What is your marital status? □ Married □ Cohabiting □ Single □ Divorced □ Widow/widower □ Other (please specify) ____________

A4. What is your educational level? □ None □ Primary school □ High school degree □ Baccalaureate □ Graduate degree □ Other (please specify) ____________

A5. What is your current job? ____________________________

A6. What is the education level of your partner? □ None □ Primary school □ High school degree □ Baccalaureate □ Graduate degree □ Other (please specify) ____________

A7. What is your partner current job? ________________________

A8. How many children do you have? ________________________

The following questions are referred to the child that you brought to the doctor.

A9. How old is your child? _____________________

A10. What is the gender of your child? □ Male □ Female

A11. What diseases does your child have? ____

A12. Does your child take any medication? □ No □ Yes

A13. How would you rate your child's health on a scale from 1 to 10, with 10 being healthiest?

1 2 3 4 5 6 7 8 9 10

B. KNOWLEDGE

The questions in this section are aimed at acquiring information on your knowledge about the recommended vaccinations for your child

B1. For which of the following diseases is available a vaccine in Italy?

Tetanus/Diphtheria/Pertussis	□ Yes	□ No	□ Do not know
Hepatitis A	□ Yes	□ No	□ Do not know
Hepatitis B	□ Yes	□ No	□ Do not know
Pneumococcal disease	□ Yes	□ No	□ Do not know
Poliomyelitis	□ Yes	□ No	□ Do not know
Seasonal influenza	□ Yes	□ No	□ Do not know
Varicella	□ Yes	□ No	□ Do not know
Measles/Mumps/Rubella	□ Yes	□ No	□ Do not know
Meningococcal disease	□ Yes	□ No	□ Do not know
Haemophilus influenzae type B	□ Yes	□ No	□ Do not know
Rotavirus infection	□ Yes	□ No	□ Do not know
Human papillomavirus (HPV) infection	□ Yes	□ No	□ Do not know

B2. Are children with chronic diseases at greater risk of complications from vaccine preventable diseases (VPDs)?

□ Yes □ No □ Do not know

B3. Which of the following vaccinations are recommended for children with chronic conditions?

□ Diphtheria	□ Tetanus	□ Pertussis	□ Hepatitis B	□ Seasonal influenza
□ Poliomyelitis	□ Measles	□ Mumps	□ Rubella	□Varicella
□ Pneumococcal	□ HPV	□ Rotavirus	□ Meningococcal	□ *Haemophilus influenzae* type B
□ Hepatitis A				

C. ATTITUDES AND BEHAVIORS

The questions in this section are aimed at acquiring information on your attitudes and behaviors towards the recommended vaccinations for your child

B1. How serious do you consider the VPDs on a scale from 1 to 10?

Not serious 1 2 3 4 5 6 7 8 9 10 Very serious

B2. How useful do you consider the vaccinations on a scale from 1 to 10?

Not useful	1	2	3	4	5	6	7	8	9	10	Very useful

B3. How dangerous do you consider the vaccinations for your child on a scale from 1 to 10?

Not dangerous	1	2	3	4	5	6	7	8	9	10	Very dangerous

B4. How worried are you about the side effects of vaccinations?

Not worried	1	2	3	4	5	6	7	8	9	10	Very worried

B5. Have you ever delayed having your child get a shot for reasons other than illness or allergy?
□ Yes, for which reason? ______________________________
□ No, for which reason? ______________________________

B6. Have you ever decided not to have your child get a shot for reasons other than illness or allergy?
□ Yes, for which reason? ______________________________
□ No, for which reason? ______________________________

B7. How sure are you that following the recommended shot schedule is a good idea for your child?

Not at all sure	1	2	3	4	5	6	7	8	9	10	Completely sure

B8. Children get more shots than are good for them.
□ Strongly disagree □ Disagree □ Not sure □ Agree □ Strongly agree

B9. I believe that many of the illnesses shots prevent are severe.
□ Strongly disagree □ Disagree □ Not sure □ Agree □ Strongly agree

B10. It is better for my child to develop immunity by getting sick than to get a shot.
□ Strongly disagree □ Disagree □ Not sure □ Agree □ Strongly agree

B11. It is better for children to get fewer vaccines at the same time.
□ Strongly disagree □ Disagree □ Not sure □ Agree □ Strongly agree

B12. How concerned are you that your child might have a serious side effect from a shot?
□ Not at all concerned □ Slightly concerned □ Not sure □ Moderately concerned □ Extremely concerned

B13. How concerned are you that any one of the childhood shots might not be safe?
□ Not at all concerned □ Slightly concerned □ Not sure □ Moderately concerned □ Extremely concerned

B14. How concerned are you that a shot might not prevent the disease?
□ Not at all concerned □ Slightly concerned □ Not sure □ Moderately concerned □ Extremely concerned

B15. If you had another infant today, would you want him/her to get all the recommended shots?
□ Yes □ No □ Do not know

B16. Overall, how hesitant about childhood shots would you consider yourself to be?
□ Not at all hesitant □ Slightly hesitant □ Not sure □ Moderately hesitant □ Extremely hesitant

B17. I trust the information I receive about shots.
□ Strongly disagree □ Disagree □ Not sure □ Agree □ Strongly agree

B18. I am able to openly discuss my concerns about shots with my child's doctor.
□ Strongly disagree □ Disagree □ Not sure □ Agree □ Strongly agree

B19. All things considered, how much do you trust your child's doctor?

Do not trust at all	1	2	3	4	5	6	7	8	9	10	Completely trust

B20. Have you ever discussed with a pediatrician/medical specialist on the recommended vaccinations for your child?
□ Yes □ No

B21. Which of the following vaccinations did your child receive?

□ Diphtheria	□ Tetanus	□ Pertussis	□ Hepatitis B	□ Seasonal Influenza
□ Poliomyelitis	□ Measles	□ Mumps	□ Rubella	□ Varicella
□ Pneumococcal	□ HPV	□ Rotavirus	□ Meningococcal B	□ Meningococcal C
□ *Haemophilus influenzae* type B		□ Hepatitis A	□ Other ______________	

B22. Who recommended you vaccinate your child?
□ No one, I decided by my self □ Pediatrician □ Medical specialist □ Other (please specify) _______

C. INFORMATION

The questions in this section are aimed to evaluate the sources from which you acquire information on the recommended vaccinations for your child

C1. From which of the following sources do you receive information about the recommended vaccinations for your child? (more than one answer is allowed)

□ None □ Pediatrician □Medical specialist □ Internet □ Mass-media □ Social media

□ Other (please specify) ____________

C2. Do you feel you need more information about the recommended vaccinations for your child? □ No □ Yes

The questionnaire is finished. Do you want to add something?

____
